# Pittsburgh Dentists

**Published:** 1852-10

**Authors:** 


### PITTSBURG DENTISTS.

We have been informed that the Profession, in Pittsburg, have formed an Association for mutual improvement. We are glad to see this spirit, and if all will enter into it with the right feeling, much good will result. By such Associations useful knowledge is disseminated, and that selfish spirit, which looks bad and is bad, is subdued, and a spirit of sociability engendered.

